# Molecular analysis indicates high levels of carabid weed seed consumption in cereal fields across Central Europe

**DOI:** 10.1007/s10340-019-01109-5

**Published:** 2019-04-09

**Authors:** Britta Frei, Yasemin Guenay, David A. Bohan, Michael Traugott, Corinna Wallinger

**Affiliations:** 1 Mountain Agriculture Research Unit, Institute of Ecology, University of Innsbruck, Technikerstraße 25, 6020 Innsbruck, Austria; 2 Agroecologie, AgroSup Dijon, INRA, Université Bourgogne Franche-Comte, 21000 Dijon, France; 3 Institute of Interdisciplinary Mountain Research, IGF, Austrian Academy of Sciences, Technikerstraße 21a, 6020 Innsbruck, Austria

**Keywords:** Carabidae, Granivory, Seed predation, Pest regulation

## Abstract

Carabid beetles are abundant in temperate agroecosystems and can play a pivotal role as biocontrol agents. While there is good knowledge regarding their effects on invertebrate pests in some systems, comparably little is known on the rate of seed feeding under field conditions. Molecular approaches are ideally suited for investigating carabid feeding interactions; to date, however, they have only been applied to animal prey. We sampled adult carabid beetles in organic cereal fields in three regions along a Central European transect. Regurgitates from populations of the three most common species, *Poecilus cupreus, Pseudoophonus rufipes* and *Pterostichus melanarius*, were screened for plant DNA, cereal aphids, collembolans and earthworms. The frequency of carabid individuals positive for plant DNA was high (> 70%) and independent of carabid species, sex, region and the time point of sampling. Detections for non-pest and pest prey were comparably lower, with 21.6% for collembolans, 18.1% for earthworms and 4.2% for aphids, respectively. Despite the prolonged detection period of plant DNA in carabid guts, as compared to animal prey, these first results suggest that weed seeds form an important part of the adult carabid diet. It would also lend support to the hypothesis that seed-feeding carabids are biocontrol agents of weeds, with effects of regulation on the weed seedbank that depend on behavioural and contextual factors including carabid species preferences for weed seed species, their life stage and tillage practices.

## Key messages

- Carabid beetles are important biocontrol agents of insect pests, which can also feed on weed seeds and alternative non-pest invertebrate prey.
- Information on the relative frequency (level) of predation within agricultural fields between these prey groups is missing
- We investigated the level of consumption of these food types in cereal crops by three common carabid beetle species across three sampling regions in Central Europe using molecular trophic approaches.
- High levels of plant DNA detection (> 70%), independent of carabid species, sex, region and season, would indicate that weed seeds are important food resources for carabids and support the hypothesis that they have the potential to regulate the weed seed bank.

## Introduction

Carabids are a diverse group of beetles that have been widely studied as biocontrol of pests such as aphids (Lang 2003; Staudacher et al. 2016; Roubinet et al. 2017), slugs (Bohan et al. 2000; Symondson et al. 2002; Thomas et al. 2009; Fusser et al. 2016; El-Danasoury et al. 2017) and a variety of other pests (Sunderland 2002). Moreover, several carabid species are known to consume substantial amounts of weed seeds (Tooley and Brust 2002; Honek et al. 2003; Talarico et al. 2016). Seeds are a food source extremely rich in many nutrients that are important to carabid development and reproduction, often equalling or even exceeding the quantities present in animal prey (Lundgren 2009). Moreover, weed seed predation has the potential to reduce the need for herbicides. Recent work on carabids in this context suggests that sufficiently high numbers of carabids could regulate weeds (Westerman et al. 2003; Bohan et al. 2011; Kulkarni et al. 2015; Quinn et al. 2016; Rusch et al. 2016; Petit et al. 2017).

Conclusions about the dietary choice of carabids within fields are mainly drawn from correlative analyses because trophic interactions among arthropods are, in general, difficult to observe (Symondson 2012). The few studies that have simultaneously monitored weed seed predation and carabid communities reveal that any observed correlations are highly variable and dependent upon the agricultural context (Saska et al. 2008; Davis and Raghu 2010; Jonason et al. 2013; Trichard et al. 2013). Conclusions from laboratory-based choice tests of carabid food preferences (Lundgren 2009) do not allow estimation of in-field frequencies of interaction (Loughridge and Luff 1983; Wallinger et al. 2015). Thus, direct detection of weed seed consumption is needed to understand seed—carabid interactions and mechanisms behind the biological control of weeds. DNA-based methods are reliable tools in trophic ecology for identifying food remains within dietary samples that cannot be assigned by traditional methods of dietary analysis. Only recently these have been used to identify consumed seeds in carabid regurgitates (Wallinger et al. 2015; Sint 2018). Diagnostic multiplex PCR allows testing for a defined set of food taxa in parallel within one single reaction and is especially useful for screening large numbers of samples, as it is both time- and cost-effective (Sint 2012). Recently, such assays have been successfully applied to prey choice in invertebrate food webs including carabids in cereal fields (Roubinet et al. 2017, 2018; Staudacher et al. 2018). In these studies, no feeding interactions were identified for about 45% of the arthropod predator individuals early in the season and 28% at the late period. An average of 38% of carabids were found not to have consumed prey that they were tested for (Staudacher et al. 2016). Similar observations have been made in a field study on the effects of fertilization on food webs in cereal fields in Austria (Manzl 2016), where about 44% of the generalist predators (72% of these were carabids) tested negative for animal prey. One potential explanation for these high fractions of carabids testing negative for animal prey is that these individuals were consuming other prey that had not been targeted in their PCR assays and in particular weed seeds. To date, however, this hypothesis of alternative prey has not been tested.

The present analysis compares the population frequencies of carabid consumption of plants, insect pests and alternative invertebrate prey. We investigated the dietary choice of adult carabids in organic cereal fields across a Central European transect, across three regions from Burgundy (France), through Tyrol (Western Austria) to the Vienna Basin (Eastern Austria). Over 1200 regurgitate samples of three highly abundant European carabid species were collected in six fields per region were analyzed. Samples were screened for DNA of plants, three species of cereal aphids and detritivorous non-pest prey groups (lumbricids, collembolans). The aim of the study was to: (i) evaluate the population level frequency of consumption of the different prey types, and to test whether the patterns are consistent among (ii) different carabid species, (iii) regions and (iv) the season.

## Materials and methods

Six organic cereal fields each, in three different regions, were selected along an east–west transect in Central Europe, comprising different climatic and ecological conditions (Frei 2018). The first region was in central Burgundy (France) near Dijon, the second in Tyrol (Western Austria) and the third in the Vienna Basin (Eastern Austria). The field sizes varied from 1 to 30 ha. Field work was conducted in 2016 during two sampling sessions: May/June and July/August. The sampling design included four, 32 m long transects per field, one on each field border, where a set of different trap types was installed at a distance of 4, 8, 16 and 32 m from the field margin. The trapping of the carabids for molecular analysis was done with dry pitfall traps, consisting of plastic funnels (Ø 7.5 cm, 11 cm in depth) with inserted plastic beakers, partly filled with wood chips and covered by metal roofs. The wood chips provided structure in the plastic pitfalls that has been found to reduce intraguild predation. Traps were kept activated for 48 h and emptied every 12 h (morning and evening). Adult carabid beetles were collected alive and put individually in reaction tubes. Beetles were stimulated to regurgitate as described in Wallinger et al. (2015), sexed, identified to species level (Müller-Motzfeld 2004) and thereafter released back into agricultural fields. Beetles that died were immediately frozen at − 24 °C to provide whole body extracts. Regurgitates were used preferentially and whole body extracts were only used where there were not enough regurgitates. We selected *Poecilus cupreus*, *Pseudoophonus rufipes* and *Pterostichus melanarius,* which are among the most dominant carabid species in European arable land (Thiele 1977; Luff 2002) and the most abundant ones in our samples. Seed feeding does occur in all three carabids (Holland 2002; Tooley and Brust 2002; Honek et al. 2007; Talarico et al. 2016), although *P. cupreus* and *P. melanarius* are described as predominantly carnivorous (Thiele 1977).

DNA extraction of the regurgitates followed Wallinger et al. (2015). Whole beetles were homogenized with glass beads (10 × Ø 3 mm and 5 × Ø 5 mm), 400 µl 1xTES buffer and 10 µl Proteinase K (20 mg/ml) via Precellys ^®^ 24 tissue homogenizer (Bertin Technologies, Montigny-le-Bretonneux, France), at 5000 rpm for 2 × 60 s prior to incubation for lysis. Samples were screened with a diagnostic multiplex PCR assay, specifically targeting DNA of the three cereal aphid species *Metopolophium dirhodum, Rhopalosiphum padi* and *Sitobion avenae*, as well as collembolans and lumbricids. For detection of weed seeds, a general plant primer pair targeting chloroplast DNA was added. The 10 µl reactions contained 2.5 µl DNA extract, 0.5 µg BSA, 5 µl 2xKAPA2G (KAPA Biosystems, Wilmington, MA, USA), 1 µl PCR-grade water and 1 µl primer mix in their respective concentrations (Table 1). The PCR conditions were: 95 °C for 3 min, followed by 35 cycles of 95 °C for 15 s for denaturation, 62.5 °C for 90 s for annealing, 72 °C for 30 s extension and 72 °C for 10 min for the final elongation. Within each PCR, there was one negative control (PCR-grade water) and one positive control (DNA mix of target species) included to check for DNA carryover contamination and amplification success. All PCR products were analysed using the QIAxcel capillary electrophoresis system (Qiagen) as described in Wallinger et al. (2015), with the exception that the method AM3201 was used.

**Table 1:** Primers used in the multiplex PCR assay for selected prey taxa. Provided are the targeted taxa/species, the original primer names, the primers’ sequences, the fragment length amplified by each primer pair, the targeted gene, the final concentration (Conc.) of each primer in the PCR and the references where primers have first been described

Target group	Primer	Sequence (5′–3′)	Fragment length (bp)	Gene	Conc. (μM)	References
Collembolan	Col3F	GGACGATYTTRTTRGTTCG	231	18 s	0.2	Kuusk and Agusti (2008)
	A415-springt	GAATTTCACCTCTAACGTCGCAG		18 s	0.2	Staudacher et al. (2016)
Lumbricids	S408-earthw	CCATGATTTCTTAGATCGTACAATCC	85	18 s	0.2	Staudacher et al. (2016)
	A413-earthw	ATARGGGTCGGAGCTTTGTG		18 s	0.2	Staudacher et al. (2016)
*Metopolophium dirhodum*	Met-dir-S436	CCTTTATCAAATAACATTGCACATAAC	105	COI	0.2	Ye et al. (2017)
	Met-dir-A440	AATAAAGTTAATTGCTCCTAAAATTGAG		COI	0.2	Ye et al. (2017)
*Rhopalosiphum padi*	Rho-pad-S440	TAATAATATAAAATTAAACCAAATTCCATTA	136	COI	0.2	Ye et al. (2017)
	Rho-pad-A442	TGATGTATTTAAATTACGATCAGTAAGAAG		COI	0.2	Ye et al. (2017)
*Sitobion avenae*	Sit-ave-S433	TCATCACTTAGAATTCTTATTCGTCTT	304	COI	0.1	Ye et al. (2017)
	Sit-ave-A438	AAGGTGGRTAAATAGTTCATCCTGTA		COI	0.1	Ye et al. (2017)
Plant	g A49425	GGGCAATCCTGAGCCAA	200	trnL	0.2	Taberlet et al. (2007)
	d B49863	GGGGATAGAGGGACTTGAAC		trnL	0.2	Taberlet et al. (2007)

Food DNA detection rates were tested for significant differences between carabid species, regions, sampling sessions and between males and females, using a generalized linear mixed model (GLMM) with the binomial distribution family and logit link. To control for false discovery due to multiple testing, Benjamini–Hochberg corrected post hoc tests were used with bound optimization by quadratic approximation. As the samples collected within fields, across transects within fields and traps within transects are not independent, a nested random effect accounting for location was introduced to the model. Differences between first versus second sampling session could only be attempted for *P.* *cupreus* because *P.* *rufipes* and *P.* *melanarius* were both absent at the first sampling session. The analysis presented for these two autumn breeders was therefore restricted to the second sampling session. Statistical analysis was conducted in R (R Core Team 2017) version 1.1–17 using the package lme4.

## Results

**Fig. 1:**
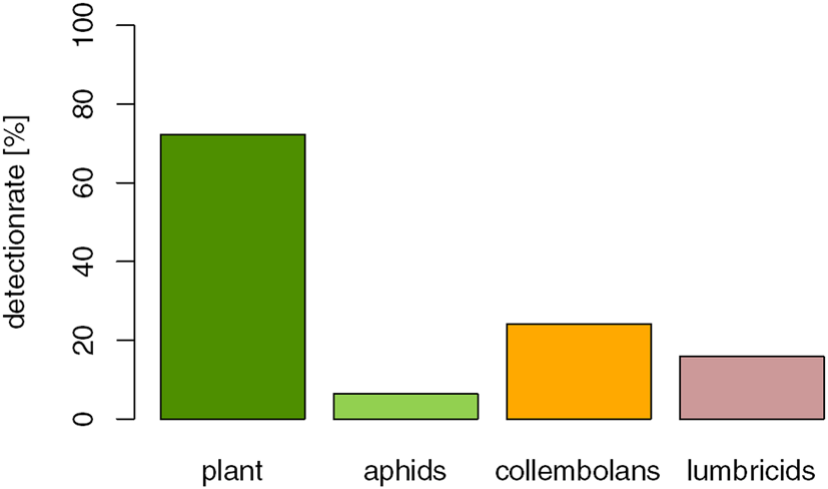
Overall detection rates of prey-specific DNA in carabid regurgitates of three carabid species: *Poecilus cupreus, Pseudoophonus rufipes* and *Pterostichus melanarius*. *Note:* As more prey types in one sample can be detected, the total sum of all columns taken together exceeds 100%

**Fig. 2:**
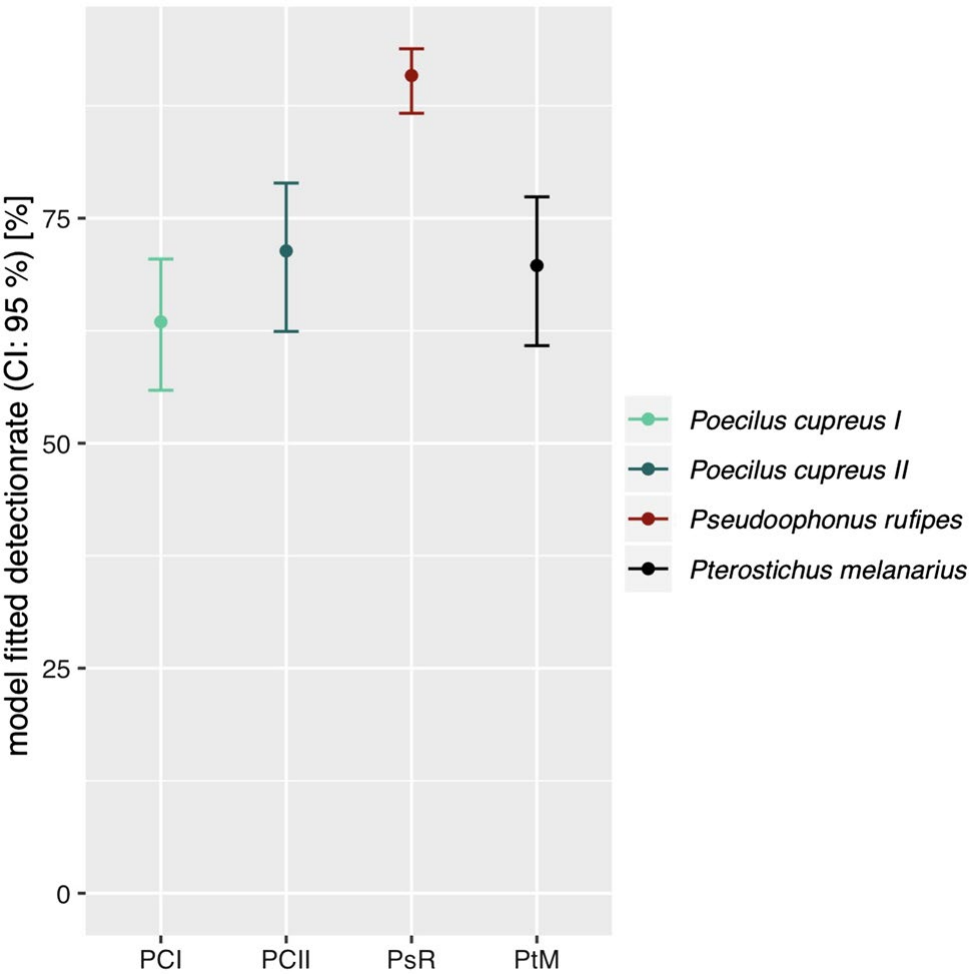
Comparison of the GLMM estimated mean plant detection rates for the three carabid species: detection rates for *P. cupreus* that have been caught in the first session have been calculated separately (*Poecilus cupreus* I) from those stemming from the second session (*Poecilus cupreus* II). *P. rufipes* and *P.* *melanarius* were present at the second sampling session only

**Fig. 3:**
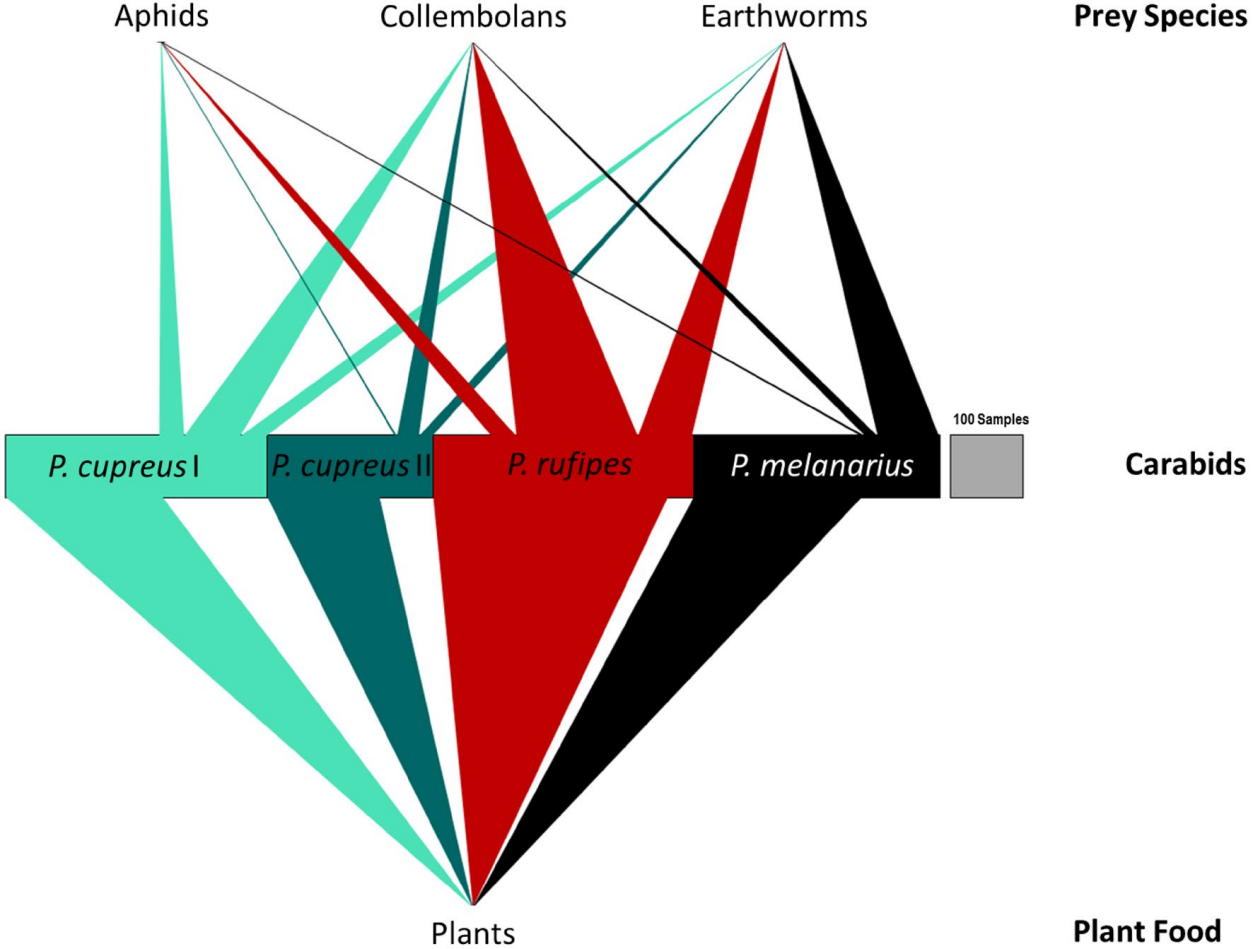
Observed prey DNA detections in regurgitates/whole body extracts of *Poecilus cupreus* at the first (*P. cupreus I*) and second sampling session (*P. cupreus* *II*), *Pseudoophonus rufipes* and *Pterostichus melanarius* across all regions. The circles stand for the different food types—i.e. aphids, collembolans and earthworms (above) and seeds (below). The width of each panel represents number of samples analysed, and width of grey boxes on the right end of each graph represents a reference number of 100 samples

Out of all 1188 samples tested (1069 regurgitates, 119 whole beetle extracts), 78% was tested positive for at least one of the targeted food types: plants, aphids, earthworms and collembolans. The model estimated detection rates for any food DNA were significantly higher in *P.* *rufipes* (94.1%) than in *P.* *melanarius* (75.1%; *p* < 0.001) and *P.* *cupreus* (75.6%, *p* < 0.001). For the comparison between carabid species, only samples from the second session were used due to the absence of the autumn breeders’ *P.* *rufipes* and *P.* *melanarius* at the first session. There was no difference in overall food detection rates between the two sampling sessions (*p* = 0.492), regions (*p* = 0.74) or beetle sexes (*p* = 0.97). All prey types tested were present in regurgitates of each carabid species and in each region. Plant detection rates were high everywhere (Fig. 1). In 49.8% of the food positive samples, plant DNA was the only food DNA detected, in 43.1% it was plant and animal, and in 7.1% it was animal DNA alone. The model-fitted plant detection rate was significantly higher in *P.* *rufipes* (90.9%, *p* > 0.001) than in *P.* *cupreus* (63.5% first and 71.4% second sampling, *p* > 0.001) and *P.* *melanarius* (69.7%, *p* > 0.001; Fig. 2). Lower detection rates were recorded for pest (aphids) and non-pest prey, i.e. lumbricids and collembolans, in comparison with plant DNA (Fig. 3). There were significantly more detections of collembolan DNA in *P.* *rufipes* (44.5%, *p* < 0.001) than in *P.* *melanarius* (4.7%, *p* < 0.001) and *P.* *cupreus* at the second sampling date (10.0%, *p* < 0.001).

## Discussion

The current study is a comparison of food DNA detection of plants, invertebrate pest and alternative prey within populations of three common carabid beetles in arable fields in three different European regions. Detection frequencies of plant DNA were high—across all regions independently of carabid species, sex and sampling time. We hypothesize that the plant DNA detected in regurgitates mainly originates from consumed seeds as has been demonstrated in numerous studies (Tooley and Brust 2002; Honek et al. 2003; Lundgren et al. 2013; Daedlow et al. 2014; Kulkarni et al. 2015; Cutler et al. 2016; Talarico et al. 2016; Birthisel et al. 2017). Our logic for this hypothesis is that while carabid beetles can eat fruit, pollen and sometimes plant leaves (Toft 2002), previous studies have shown that herbivory (i.e. the consumption of other plant tissue than seeds) is negligible (Goldschmidt and Toft 1997). Non-reproductive plant tissue is a poor food source, being especially low in nitrogen and often containing toxic secondary compounds, in comparison with seeds. Fruits can be excluded as carabid food source in the current study, due to the absence of fruit-bearing plants in the fields at sample collection. To minimize the risk of false positives resulting from potential DNA carryover via pollen that might have been attached to the carabids surface, we have chosen a molecular marker targeting the *trn*L-region, a part of chloroplast DNA (cpDNA), which rarely is present in pollen. cpDNA is primarily inherited maternally and therefore a widely accepted molecular marker for seeds (McCauley et al. 2007). The high detection rates of cpDNA in field-collected regurgitates is therefore likely to be due to plant seeds, and more specifically the seeds of weeds (i.e. non-crop plants) because crop seeds were absent during the sampling periods. Admittedly, there were also some seed-bearing plants at the field margins which could have been additionally eaten by carabids caught in the outer traps. To gain more insight on this, we are currently identifying the consumed seeds on a species-specific level via a metabarcoding approach.

Although it was already known that many carabids do consume seeds, the high proportion of field-caught beetles that were positive for plant DNA was striking. While the relative frequencies of seed consumption may be overestimated due to longer post-feeding detection intervals of plant food compared to animal prey (Staudacher et al. 2011; Sint 2018), our findings lend support to the hypothesis that seed-feeding adult carabids are significant consumers of weed seeds (Bohan et al. 2011; Kulkarni et al. 2015; Quinn et al. 2016; Rusch et al. 2016; Petit et al. 2017).

The examination done here is restricted to adult beetles because soil-living carabid larvae are poorly trapped by the pitfall trap methodology we employed. Based on laboratory studies, carabid larvae have been reported to consume substantial amounts of seeds. Some species including *P.* *rufipes* are known to build seed caches (Thiele 1977; Hartke et al. 1998; Lundgren et al. 2009), and some carabid larvae require weed seeds to complete their development (Saska and Jarosik 2001; Saska 2005, 2015). Future studies may shed light into the seed consumption of field-collected larvae using molecular approaches.

Among the populations of the three carabid species, *P.* *rufipes* showed an especially high frequency of detection for seeds. This finding is in accordance with the prior trophic characterization of this carabid species as a seed predator (Thiele 1977; Holland 2002) and laboratory feeding experiments that indicate that seeds are their preferred food source (Toft 2002; Cutler et al. 2016).

All food types that were present in populations of each carabid species in each region fit with their omnivorous feeding nature and confirm previous studies revealing the broad diet range of the three species (Toft 2002; Lundgren et al. 2009). The 22% of samples without any food detection were predominantly *P.* *cupreus* and *P.* *melanarius* (only 6% of these negatives were *P.* *rufipes*). Our multiplex PCR system did not target all possible prey, and these beetles may have consumed other prey food items. Given that *P.* *cupreus* and *P.* *melanarius* also showed significantly less prey detection in general, it is likely that we missed certain prey types that are important food, i.e. dipterans, thrips and intraguild prey, such as other carabid species or spiders. Low levels of predation on aphids, in comparison with other studies in European cereal fields (Staudacher et al. 2016; Roubinet et al. 2017, 2018), may be ascribed to the fact that there were only few aphids present in the sampled fields. Spontaneously high detections of aphids as food in individual fields would tend to support their potential as aphid biocontrol agents (Harwood and Obrycki 2005). Overall detection rates of lumbricid and collembolan alternative prey were higher than in Staudacher et al. (2016) and lower than in Roubinet et al. (2017, 2018). However, in concurrence with our results, these studies revealed a preference for lumbricids over collembolans in *P.* *cupreus* and *P.* *melanarius* and the reverse preference in *P.* *rufipes*.

For biocontrol, consumption rates are a key variable of interest. This parameter represents the amount of food taken in relation to its availability in the field, per standardized unit of time. Therefore, comparing molecular prey detection rates with prey availability is the next logical development of our work. However, it is not straightforward to quantify predation rates by molecular gut content analysis (Birkhofer et al. 2017). Still, the frequency of detecting specific food taxa in a large sample of consumers can provide a good proxy for the strength of specific trophic interactions and also for the amount of food consumed (Baker et al. 2014; Deagle 2018). Based on this, our findings provide a strong indication that seeds are highly frequently consumed by the investigated carabid species, potentially surpassing the consumption of animal prey.

## Conclusion

The present study contributes to a better understanding of the consumption of plant and animal prey by three dominant carabid species in European arable land. By revealing high frequencies of plant consumption in cereal fields across Central Europe, our findings point to the importance of weed seeds in the diet of adult carabids. The gut content analyses also corroborate recent field studies demonstrating the importance of carabids as biocontrol agents not only for invertebrate pests but also for weeds. In the future, more insight is needed into the magnitude and importance of the effect and finding agricultural management practices that enhance or augment carabid weed seed predation and regulation.

## Author contributions

M.T., D.B. and C.W. conceived and designed the study. C.W., M.T., B.F., Y.G. and D.B. performed the field sampling. Y.G. and B.F. were responsible for the molecular work. B.F analysed the data and contributed together with Y.G. to the first draft. C.W. wrote the manuscript, which all authors revised and finally approved to be published.
